# Unlocking the Door to Neuronal Woes in Alzheimer’s Disease: Aβ and Mitochondrial Permeability Transition Pore

**DOI:** 10.3390/ph3061936

**Published:** 2010-06-14

**Authors:** Heng Du, Shirley ShiDu Yan

**Affiliations:** 1 Department of Surgery, Physicians & Surgeons College of Columbia University, New York, NY 10032, USA; 2 Department of Pathology & Cell Biology, Physicians & Surgeons College of Columbia University, New York, NY 10032, USA; 3 The Taub institute for Research on Alzheimer’s Disease and the Aging Brain, Columbia University, New York, NY 10032, USA

**Keywords:** amyloid beta, mitochondrial permeability transition, cyclophilin D, therapy

## Abstract

Mitochondrial dysfunction occurs early in the progression of Alzheimer’s disease. Amyloid-β peptide has deleterious effects on mitochondrial function and contributes to energy failure, respiratory chain impairment, neuronal apoptosis, and generation of reactive oxygen species in Alzheimer’s disease. The mech**a**nisms underlying amyloid-β induced mitochondrial stress remain unclear. Emerging evidence indicates that mitochondrial permeability transition pore is important for maintenance of mitochondrial and neuronal function in aging and neurodegenerative disease. Cyclophilin D (Cyp D) plays a central role in opening mitochondrial permeability transition pore, ultimately leading to cell death. Interaction of amyloid-β with cyclophilin D triggers or enhances the formation of mitochondrial permeability transition pores, consequently exacerbating mitochondrial and neuronal dysfunction, as shown by decreased mitochondrial membrane potential, impaired mitochondrial respiration function, and increased oxidative stress and cytochrome c release. Blockade of cyclophilin D by genetic abrogation or pharmacologic inhibition protects mitochondria and neurons from amyloid-β induced toxicity, suggesting that cyclophilin D dependent mitochondrial transition pore is a therapeutic target for Alzheimer’s disease.

## 1. Introduction

Amyloid beta (Aβ), a major component of amyloid plaque, is a neurotoxic peptide, the accumulation of which leads to neuronal degeneration relevant to the pathogenesis of Alzheimer’s disease (AD) [1,2,3,4,5]. Aβ accumulates in the extracellular and intracellular compartments, including mitochondria. Notably, recent studies from several independent groups including our laboratory demonstrate the accumulation of Aβ in mitochondria of brains from AD patients and AD mouse models [1,6,7,8,9,10,11,12]. It is known that progressive accumulation of mitochondrial Aβ is significantly related to the mitochondrial and neuronal dysfunction in an Aβ rich environment [1,6,7,11,12,13,14]. Predominant mitochondrial pathological changes in AD include mitochondrial membrane potential dissipation [15,16,17], respiration defect [6,7,18], oxidative stress [1,14,19,20,21], Aβ accumulation in mitochondria [1,5,6,10,11], impaired calcium buffering capacity [22,23,24,25], altered mitochondrial dynamics and trafficking [26,27,28], mtDNA mutation [29,30,31] and mitochondrial permeability transition [7,13,32]. Mitochondria are essential for provision of energy by oxidative phosphorylation; this organelle also modulates intra-neuronal calcium homeostasis necessary to sustain neuronal function and survival. Dysregulation of mitochondrial function leads to synaptic stress, disruption of synaptic transmission, apoptosis and ultimately neuronal death [6,15,33,34,35]. Thus, it is highly important to unravel the mechanism(s) of Aβ-associated mitochondrial alterations to enhance our understanding of the pathophysiological process of AD.

Recent studies emphasize that mitochondrial permeability transition pore (mPTP) is involved in Aβ induced mitochondrial perturbation [7,13,25,32,36,37,38]. The formation of mPTP is closely related to Aβ superimposition and perturbation of mitochondrial structure and function. Inhibition of mPTP formation in an AD animal model and in Aβ-insulted cells results in enhanced protection of neurons from Aβ toxicity and oxidative stress. Here, we review the role of mPTP in mitochondrial pathology relevant to the pathogenesis of AD, particularly related to the involvement of Cyclophilin D (Cyp D) in mPTP. 

## 2. mPTP and Alzheimer’s Disease

Mitochondrial permeability transition, or MPT, is an increase in permeability of the mitochondrial membranes to molecules of less than 1,500 Daltons in molecular weight. MPT results from opening of mitochondrial permeability transition pores, known as MPT pores or mPTP. mPTP is a protein pore that is formed in mitochondrial membranes under certain pathological conditions such as oxidative stress, ischemia, traumatic brain injury and stroke. Induction of the permeability transition pore can lead to mitochondrial swelling and cell death. The deleterious impact of mitochondrial permeability transition on mitochondrial function has long been proposed [39,40,41]. 

Mitochondria are two-membrane encapsulated organelles with strict regulation of the uptake and release of substances. Disruptions in this regulation lead to mitochondrial and cellular perturbation. For example, the release of cytochrome c from mitochondria triggers a signal transduction cascade and apoptosis. mPTP is one among several factors that interfere with the integrity of mitochondrial membrane. The formation of mPTP in a mitochondrial membrane opens a nonselective portal that results in abnormal exchange of solutes and molecules > 1,500 Daltons between mitochondria and cytoplasm [42]. 

Though the exact structure of the mPTP is still unknown, it is postulated that several proteins come together to form the pore, including the outer membrane voltage-dependent anion channel (VDAC), adenine nucleotide translocase (ANT) located in the mitochondrial inner membrane, and cyp D residing in mitochondrial matrix [43,44,45,46]. A recent study suggests that phosphate carrier (PiC) in mitochondrial inner membrane is also a possible component of mPTP [47]. It is known that formation of mPTP relies on the translocation of cyp D to inner mitochondrial membrane and the intra- mitochondrial perturbations of calcium, phosphate and oxidative stress are strong inducers of the cyp D translocation [39,42,47,48,49]. Mitochondria undergoing mPTP show dissipated membrane potential, perturbed mitochondrial respiration chain, decreased ATP production, increased free radical generation, and disruption of calcium modulation [7,40,50]. Calcium effluxes from mitochondria while cytoplasm solutes flow into mitochondria; thereby causing mitochondrial swelling that in turn leads to ruptures in mitochondrial membrane. Importantly, pre-apoptotic molecules such as cytochrome c are released from the mPTP afflicted mitochondria through these ruptures, which then trigger apoptosis [43,50]. Obviously, mPTP formation is a detrimental process that significantly contributes to mitochondrial and cellular malfunction.

Involvement of mPTP in neurodegeneration has been reported in neurodegenerative diseases, including AD [7], ALS (amyotrophic lateral sclerosis) [51,52], HD (Huntington’s disease) [53] and PD (Parkinson’s disease) [54,55], as evidenced by increased CypD expression, decreased mitochondrial calcium handling capacity and mitochondrial oxidative stress in disease-affected brain regions. In our published studies, we demonstrated that CypD levels were elevated in mitochondria isolated from the hippocampus and temporal pole of AD patients. Increased Cyp D expression is predominantly localized in neurons in these specific areas of AD patients [7]. Given the positive correlation of Cyp D expression to mPTP opening [7,43,52,56], neurons with increased expression of CypD in AD-affected brain regions would be more susceptible to mPTP formation and the resultant consequences. Similarly, AD mice overexpressing amyloid precursor protein (APP) and Aβ (APP mice) demonstrated up-regulation of CypD expression in cortical mitochondria. As expected, cortical mitochondria containing Aβ undergo increased mitochondrial swelling in the presence of calcium. In addition, APP mice demonstrate increased CypD translocation to mitochondrial inner membrane and decreased mitochondrial calcium buffering capacity, suggesting that mitochondria enriched for Aβ environment are susceptible to mPTP formation, which is consistent with increased CypD expression also seen in this strain [7,13]. 

Transgenic AD mouse models show age-dependent accumulation of cerebral/mitochondrial Aβ as well as neuronal and mitochondrial stress. In APP mice (J-20 line), Aβ accumulation in the brain occurs by 4-5 months and progresses with age. By the age of 10-12 months, there are plentiful amyloid deposits in the brain [57,58]. Consistent with this observation, impaired mPTP function and calcium buffering capacity correlate with age-related Aβ accumulation in APP mouse brain and mitochondria. Cortical mitochondria from transgenic and nonTg mice showed swelling in response to Ca^2+^, although APP mitochondria show greater swelling compared to nonTg mitochondria at the ages of 12-24 months. Cortical mitochondria of both nonTg and APP mice exhibited an age-dependent increased swelling in response to Ca^2+^. Similarly, brain mitochondria isolated from APP mice demonstrated an age-related mitochondrial respiration defect, mitochondrial oxidative stress, and decreased ATP production [7,13]. Another study showed that the inhibition of ANT substantially attenuated apoptosis and autophagy in a mouse model for cerebral amyloid angiopathy, lending further credence to the involvement of mPTP in AD [59]. Taken together, these data indicate that mitochondrial permeability transition pore is sensitized in the Aβ milieu.

## 3. The Interplay of Aβ and mPTP

Aβ has been shown to directly perturb mPTP function. Moreira and colleagues demonstrated that Aβ directly induces mitochondrial swelling, cytochrome c release and mitochondrial membrane potential decrease in isolated brain mitochondria [37,60]. Administration of Aβ 25-35 triggers mPTP formation accompanying mitochondrial oxidative stress [38]. We observed in isolated brain, that the addition of Aβ to mitochondria in the presence of mPTP inducers (e.g. phosphate) enhances mitochondrial swelling in a dose-dependent manner [7]. It has been demonstrated that Aβ treatment significantly sequestrates Cyp D translocation to mitochondrial inner membrane and reduces mitochondrial calcium buffering capacity. These data indicate that Aβ is responsible for mPTP formation. 

An indirect effect of Aβ on mPTP is due to the ability of Aβ to elevate intra-cellular calcium and free radical levels. Aβ, a peptide cleaved from its precursor protein (APP), causes severe intra-neuronal free radical injury and calcium dysregulation, leading to accelerated neuronal damage [22,61,62,63]. Calcium and free radicals are strong inducers of mPTP and conversely mPTP formation further exacerbates calcium perturbation and oxidative stress. Thus, it is proposed that deregulated neuronal calcium metabolism and accumulation/production of reactive oxygen species (ROS) are possible mechanisms underlying Aβ-induced mPTP formation. Aβ treatment in cells or primary cultured neurons induces oxidative stress, calcium perturbation, increased cobalt quenching of intra-mitochondrial calcein intensity and mitochondrial cytochrome c release, suggesting the involvement of mPTP formation in an Aβ-induced disturbance of calcium and free radical production [60,64,65,66,67,68,69,70]. Thus, Aβ mediates ROS accumulation and stimulates intracellular and intra-mitochondrial calcium accumulation, thereby triggering the formation of mPTP, which, in turn, leads to further mitochondrial calcium efflux and free radical generation from mitochondria.

Mechanistically, we know that Aβ enhances translocation of CypD to mitochondrial inner membrane to trigger mPTP formation and forming Aβ-Cyp D complex. Using co-immunoprecipitation of Aβ and Cyp D, Aβ-Cyp D complex was found in mitochondrial fractions from brains of AD subjects and APP mice as well as in neurons and isolated brain mitochondria exposed to Aβ. These findings indicate the presence of Aβ-CypD interaction *in vivo* in brain mitochondria. Using surface plasmon resonance (SPR), different species of Aβ, including monomeric and oligomeric Aβ, were found to have high affinity for binding to Cyp D *in vitro*, confirming interaction of Aβ with Cyp D [7,13]. In addition, a recent report using molecular docking experiments postulates that Aβ binds with ANT [71]. However, we are not aware of any conclusive report regarding interaction of Aβ with ANT.

Although Aβ or oxidative stress could directly or indirectly affect mitochondrial function, such as mPTP formation, enhancement of mPTP formation by Aβ might be due to synergistic action of the two. Given that mPTP is critical for mitochondrial pathology and neuronal dysfunction in the pathogenesis of AD, blocking or limiting mPTP formation holds potential as a therapeutic strategy for AD. 

## 4. Blockage of mPTP Attenuates Aβ-Mediated Neuronal and Mitochondrial Malfunction

Several studies have shown that the blockage of mPTP by either genetic depletion of the mPTP key component, Cyp D or through use of the Cyp D or VDAC inhibitors protects neurons against oxidative stress- or Aβ-induced injury [7,72,73,74]. Genetic depletion of Cyp D decreases mitochondrial swelling induced by calcium. Cyp D deficient cells show less oxidative stress and apoptosis and maintain mitochondrial membrane potential even in the presence of stress inducers [7,43,50]. Further, Cyp D depletion attenuates cardiac ischemia and reperfusion injuries in mice [7,75,76] and ameliorates axonal degeneration and movement disorders in a multiple sclerosis (MS) mouse model [77]. To investigate the protective effect of blockading mPTP by genetic depletion of Cyp D in an Aβ milieu, we generated Cyp D deficient APP mice by crossing APP/Aβ overexpressing mice (APP mice) to Cyp D-deficient mice and then investigated the effect of Cyp D depletion on Aβ-induced toxicity. Cyp D-deficient APP mice preserve mitochondrial function including mitochondrial cytochrome c oxidase activity, mitochondrial respiration control ratio and mitochondrial ATP production. Furthermore, the protective effects of CypD deficiency were observed even in aged AD mice (22–24 months), suggesting that abrogation of CypD results in persistent life-long protection against Aβ toxicity in an Alzheimer’s disease mouse model [7,13]. Cyp D depletion also results in improved synaptic function and spatial learning memory, even in aged 22-24-month-old APP mice.

Pharmaceutical inhibition of Cyp D is another approach to inhibit mPTP formation. There are several known Cyp D inhibitors: cyclosporin A, sanglifehrin A, FK506 and FK1706. Administration of these inhibitors results in significant protection against mPTP-associated mitochondrial pathology in several animal models of neurodegenerative diseases, as follows. Cyclosporin A injection ameliorated the moving disorders in an ALS mouse models [78]. Cyclosporin A, FK506 or FK1706 treatment attenuated the symptoms of MS mouse models [79]. Administration of cyclosporin A or FK506 also had protective effects on HD mouse model [80]; the protective effects of these Cyp D inhibitors are proposed to be, at least in part, due to the inhibition of mPTP formation. Notably, treatment with Cyp D inhibitors at experimental dosages did not show detectable adverse effects in the mice, suggesting probable safety of these drugs in clinical translation. 

The effect of Cyp D inhibitors on Aβ toxicity has also been investigated. We and other groups have demonstrated that cyclosporin A significantly inhibited apoptosis and production of oxidative stress induced by Aβ accumulation [7,13,32,81]. Cyclosporin A treatment attenuated Aβ- induced mitochondrial swelling and increased mitochondrial calcium buffering capacity. In addition, the addition of cyclosporin A to the hippocampal CA1 region completely rescued Aβ-induced long term potentiation (LTP) reduction. These data indicate that pharmaceutical inhibition of Cyp D is a potential strategy to protect neurons from Aβ toxicity [7]. It remains to be determined whether the protective effects of CypD inhibitors are present in animal model studies. 

VDAC is another key component of mPTP. A recent report using a VDAC inhibitor, cholest-4-en-3-one oxime (TRO19622), showed results of significantly extended lifespan as well as attenuation of symptoms in G93A SOD1 ALS mice [82]. TRO19622 has not yet been tested in AD mouse or cell models. 4,4’-Diisothiocyanatostilbene-2,2’-disulfonic acid (DIDS) is a VDAC blocker and has been shown to protect cells from VDAC -potentiated cell apoptosis [73]. Small and his colleagues demonstrated that DIDS protected against neurotoxicity induced by Aβ25-35 or staurosporine on primary cultured neurons as evidenced by significantly less cell death upon the application of DIDS. These findings implicate that inhibiting VDAC-mediated mPTP might be a potential therapeutic option for the protection of neurodegeneration in AD [74].

In summary, interventions affecting mPTP formation such as genetic Cyp D depletion or use of Cyp D or VDAC inhibitors have been proven experimentally to be effective in counteracting the detrimental effects of Aβ or oxidative stress on mitochondrial and neuronal perturbation, suggesting that targeting mPTP may result in the rescue of neurons from Aβ-induced damage. 

## 5. Conclusions

**Figure 1:**
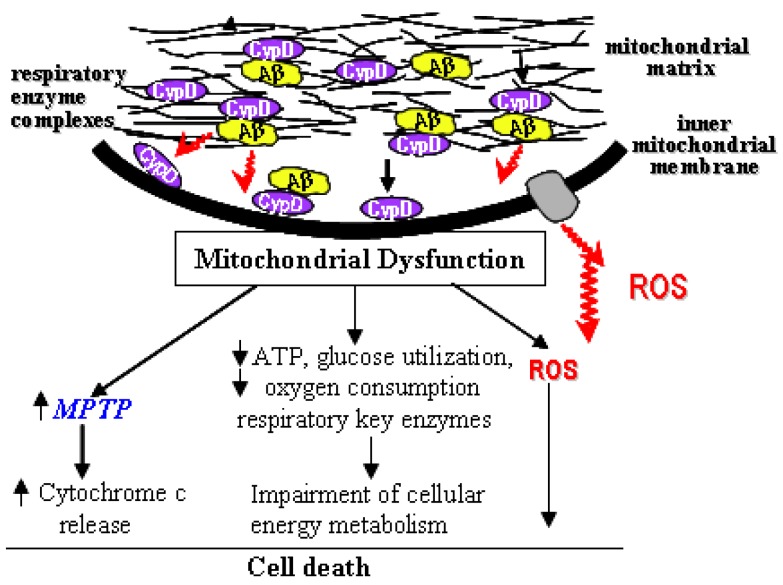
Cyclophilin D-Aβ interaction: implications for mitochondrial function.

We reviewed here the recent studies eliciting the involvement of mPTP in the pathogenesis of AD and the effects of inhibiting mPTP on mitochondrial and neuronal dysfunction. The interplay of Aβ with mPTP may be a novel mechanism underlying Aβ-associated mitochondrial pathology. CypD-dependent mitochondrial permeability transition contributes significantly to Aβ-induced neuronal and mitochondrial injury relevant to the pathogenesis of Alzheimer’s disease. In an Aβ-rich environment, Aβ gains access to the mitochondrial matrix by the translocase of the outer membrane (TOM) machinery [10] or an as yet unknown mechanism, and forms a complex with CypD, promoting its translocation to the inner mitochondrial membrane and formation of mPTP. In addition, CypD-Aβ interaction enhances generation of ROS and triggers signal transduction. These events eventually lead to cell death relevant to the AD pathogenesis (Figure 1). Thus, decreasing CypD dependent mPTP formation through pharmacologic inhibition on cyp D is an important therapeutic target for prevention and treatment of Alzheimer disease and other neurodegenerative diseases. There is also potential benefit in the development of new inhibitors of other mitochondrial transition pore components (e.g. VDAC) as therapeutic approaches to treat AD and other diseases.
